# A Novel Hybrid Iron Regulation Network Combines Features from Pathogenic and Nonpathogenic Yeasts

**DOI:** 10.1128/mBio.01782-16

**Published:** 2016-10-18

**Authors:** Franziska Gerwien, Abu Safyan, Stephanie Wisgott, Fabrice Hille, Philipp Kaemmer, Jörg Linde, Sascha Brunke, Lydia Kasper, Bernhard Hube

**Affiliations:** a Leibniz Institute for Natural Product Research and Infection Biology, Hans Knoell Institute, Department of Microbial Pathogenicity Mechanisms, Jena, Germany; b Leibniz Institute for Natural Product Research and Infection Biology, Hans Knoell Institute, Research Group Systems Biology and Bioinformatics, Jena, Germany; c Friedrich Schiller University, Jena, Germany; d Center for Sepsis Control and Care (CSCC), University Hospital Jena, Jena, Germany

## Abstract

Iron is an essential micronutrient for both pathogens and their hosts, which restrict iron availability during infections in an effort to prevent microbial growth. Successful human pathogens like the yeast *Candida glabrata* have thus developed effective iron acquisition strategies. Their regulation has been investigated well for some pathogenic fungi and in the model organism *Saccharomyces cerevisiae*, which employs an evolutionarily derived system. Here, we show that *C. glabrata* uses a regulation network largely consisting of components of the *S. cerevisiae* regulon but also of elements of other pathogenic fungi. Specifically, similarly to baker’s yeast, Aft1 is the main positive regulator under iron starvation conditions, while Cth2 degrades mRNAs encoding iron-requiring enzymes. However, unlike the case with *S. cerevisiae*, a Sef1 ortholog is required for full growth under iron limitation conditions, making *C. glabrata* an evolutionary intermediate to *SEF1*-dependent fungal pathogens. Therefore, *C. glabrata* has evolved an iron homeostasis system which seems to be unique within the pathogenic fungi.

## IMPORTANCE

The fungus *Candida glabrata* represents an evolutionarily close relative of the well-studied and benign baker’s yeast and model organism *Saccharomyces cerevisiae*. On the other hand, *C. glabrata* is an important opportunistic human pathogen causing both superficial and systemic infections. The ability to acquire trace metals, in particular, iron, and to tightly regulate this process during infection is considered an important virulence attribute of a variety of pathogens. Importantly, *S. cerevisiae* uses a highly derivative regulatory system distinct from those of other fungi. Until now, the regulatory mechanism of iron homeostasis in *C. glabrata* has been mostly unknown. Our study revealed a hybrid iron regulation network that is unique to *C. glabrata* and is placed at an evolutionary midpoint between those of *S. cerevisiae* and related fungal pathogens. We thereby show that, in the host, even a successful human pathogen can rely largely on a strategy normally found in nonpathogenic fungi from a terrestrial environment.

## INTRODUCTION

Iron is an essential micronutrient for all living organisms. Usually incorporated into heme or bound in iron sulfur clusters, it acts as an important cofactor for various cellular processes, including the tricarboxylic acid (TCA) cycle, DNA replication, chromatin remodeling, mitochondrial respiration, and detoxification of reactive oxygen species (ROS) (1). Its biological functions derive from its redox properties, based on the switch between the ferric state (Fe^3+^) and the ferrous state (Fe^2+^). This switch can, however, also become detrimental to the cell. While Fe^3+^ is poorly soluble under alkaline conditions, Fe^2+^ can become toxic by promoting the production of ROS via the Fenton reaction (2). Thus, uptake, distribution, utilization, and storage of iron have to be tightly regulated. In terrestrial environments, the poor solubility of Fe is the main reason for its low bioavailability (1). During infection, pathogenic microbes similarly face iron limitation mainly due to “nutritional immunity”—a process by which the host restricts iron to prevent proliferation of invading pathogens (3). In the human body, for example, iron is sequestered by various carrier and storage proteins such as hemoglobin, transferrin, and ferritin, and there is virtually no freely bioavailable iron (4). Therefore, successful pathogens had to develop sophisticated mechanisms to exploit host iron sources.

One of these is *Candida glabrata*, an opportunistic fungal pathogen that colonizes epithelial surfaces like the gastrointestinal tract as part of the normal human microbiota (5). However, under certain predisposing conditions, like immunosuppression or long-term hospitalization, the fungus can shift from commensalism to a pathogenic lifestyle. *C. glabrata*-induced diseases can range from superficial oral infections to systemic infections with high mortality rates (6). Among *Candida* species, *C. glabrata* ranks second to *Candida albicans* in isolation frequency in the United States and most of Europe, and together, those two species are responsible for 65% to 75% of all life-threatening systemic candidiasis (5, 7). Still, these two pathogens differ remarkably in their lifestyle, genetic makeup, and morphology (8). In fact, *C. glabrata* is much more closely related to the normally nonpathogenic baker’s yeast *Saccharomyces cerevisiae* than to *C. albicans* (9), and their respective pathogenicity strategies must have evolved independently.

A widespread strategy of pathogens, and an important virulence attribute for several fungal pathogens, is efficient and tightly controlled iron utilization. Fungi as diverse as *C. albicans*, *Histoplasma capsulatum*, *Aspergillus fumigatus*, and *Cryptococcus neoformans* harbor complex iron uptake and homeostasis mechanisms for their survival in the host (10–13). Frequently, high-affinity (HA-) uptake systems allow transport via the fungal membrane under iron starvation conditions (14), and siderophores are produced by many fungi, but not by *Candida* spp., which instead possess an uptake system for xenosiderophores (1, 15). Finally, host iron sources can be exploited: *C. albicans*, for example, uses many host iron sources like hemoglobin, ferritin, or transferrin (15–18), but no such uptake systems are known in *C. glabrata* (or *S. cerevisiae*) (15, 17, 18). In *C. albicans*, as in most other fungi, regulation of iron homeostasis is tightly controlled and mediated by two reciprocally acting transcriptional repressors, a GATA-type factor, Sfu1, and a CCAAT-binding factor, Hap43 (19). Under iron restriction conditions, the Cys_6_Zn_2_ transcriptional activator Sef1 induces iron uptake genes, while Hap43 represses iron-consuming processes as well as transcription of Sfu1. Under iron-replete conditions, lack of Sef1 activation leads to the absence of Hap43 and hence to an induction of Sfu1, which represses iron uptake (20). *S. cerevisiae* employs an iron regulatory system completely different from those employed by other fungi. Here, Aft1 is the major activator of iron uptake and recycling under iron restriction conditions (21, 22), together with its paralog Aft2, which contributes in a largely redundant manner (23). Additionally, posttranscriptional degradation of specific mRNAs ensures the downregulation of iron-consuming processes (24). Although an ortholog of Sef1 is present in *S. cerevisiae*, no iron-related function has been described for it. Both *HAP43* and *SFU1* are absent in the yeast genome.

In the present study, we analyzed *C. glabrata* mutants with deletions of potential orthologs of iron regulatory systems of both *C. albicans* and *S. cerevisiae*. Our experiments identified Aft1 as the main regulator for iron uptake and recycling under iron deprivation conditions and the mRNA degrading protein Cth2 as a posttranscriptional inhibitor of iron consumption processes. Surprisingly, the *SEF1* transcript was found to be a novel target of Cth2 with importance for growth under iron limitation conditions in *C. glabrata*. We show that *C. glabrata* employs a unique iron regulatory system, containing elements of both *S. cerevisiae* and *C. albicans* and differing from other pathogens.

## RESULTS

### Screening of a mutant library under iron restriction conditions identified *C*. *glabrata* transcription factors Aft1 and Sef1.

**FIG 1:**
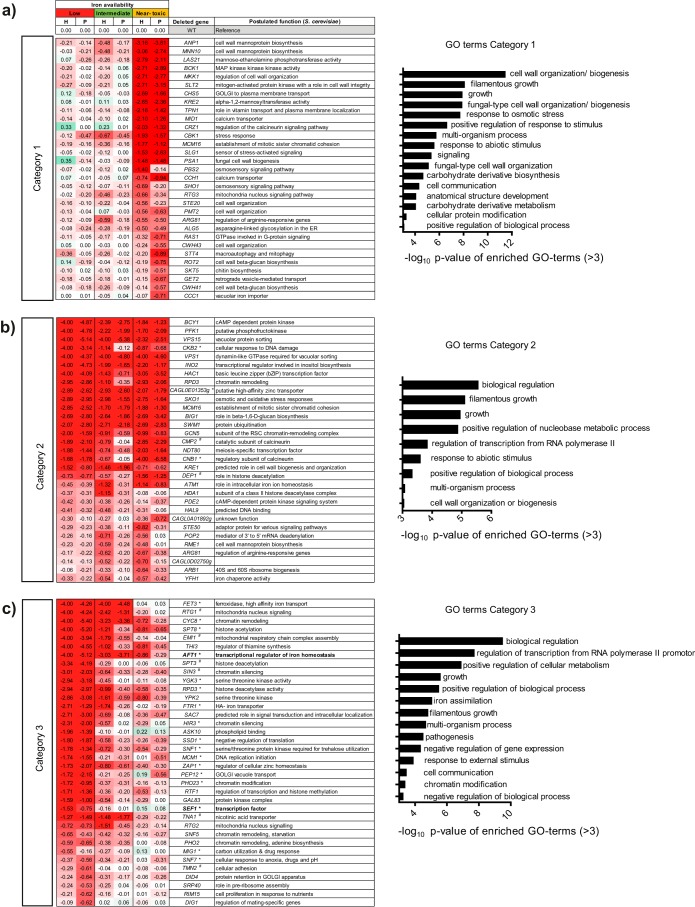
Screen-revealed *C. glabrata* deletion mutants with iron-dependent growth defects. Growth of iron-prestarved cells was assayed by measuring the OD_600_ under low-iron conditions (10 µM FeCl_3_), intermediate-iron conditions (500 µM FeCl_3_), and near-toxic iron conditions (40 mM FeCl_3_) in Chelex-treated SD (pH 5.8). Growth was evaluated compared to the WT by assessing the time of half-maximal growth (H) and the height of the plateau (P) after 50 h at 37°C. Zero corresponds to wild-type (WT) growth (shaded white), positive numbers indicate improved growth (shaded green), and negative numbers indicate growth defects (shaded red). Means of results of triplicate experiments are shown with growth equal to or less than a set cutoff of −0.5 for at least one evaluated parameter. Enriched GO-terms (–log_10_ *P* value of >3 displayed) are shown. Selected mutants were tested separately for growth on solid medium containing the iron chelator BPS. Of 27 tested mutants, 19 showed a growth defect (*), while 8 grew as well as the WT (^#^). (a) List of mutants with a growth defect under near-toxic iron conditions (category 1). (b) List of mutants with general growth defects under all tested iron conditions (category 2). (c) List of mutants with an iron-dependent growth defect that can be partially rescued by iron addition (category 3). Potential iron regulators are labeled in bold. cAMP, cyclic AMP; RSC, remodels the structure of chromatin; MAP, mitogen-activated protein kinase.

To identify factors important for iron homeostasis, an extensive deletion mutant library (Schwarzmüller et al. [25] and mutants generated in this work) was screened for iron-dependent growth defects. In total, 649 strains were analyzed for their growth behavior under conditions of different iron concentrations, and 100 of them displayed an iron-dependent phenotype under at least one set of conditions. These strains were divided into three different categories according to their growth behavior (Fig. 1). Category 1 contained 31 mutants with growth defects under specifically near-toxic iron conditions (Fig. 1a). Gene ontology (GO)-term analysis revealed an enrichment of processes associated with cell wall organization and integrity in this category. A mutant lacking Ccc1, a transporter with a possible role in vacuolar iron sequestration (26), also appeared in this category, while its supposed regulator Yap5 (27) seemed to play no role (see Dataset S1 in the supplemental material). Category 2 contained another 31 mutants with general growth defects that were independent of iron supplementation (Fig. 1b), including deletions of known and iron-associated genes (*YFH1*, *ATM1*) conserved in most fungi such as *C. albicans* (28, 29) and *S. cerevisiae* (30, 31). Additionally, category 1 and 2 contained strains lacking genes encoding components of calcium signaling or the stress-associated calcineurin pathway (*CCH1*, *MID1*, *CMP2*, *CNB1*, *CRZ1*). Most relevant to this work, category 3 contained 36 mutants with growth defects under low-iron conditions that were rescued by iron addition (Fig. 1c). GO-term analysis revealed an enrichment of genes involved in transcription regulation, chromatin remodeling (*CYC8*, *PHO23*), and—as expected—iron assimilation. These included the iron acquisition genes *FTR1* and *FET3* as well as *AFT1* and *SEF1*, encoding orthologs of activators of iron acquisition in *S. cerevisiae* and *C. albicans*, respectively.

### *AFT1* and *SEF1*, but no other known genes encoding iron regulator orthologs, are required under iron limitation conditions.

**FIG 2:**
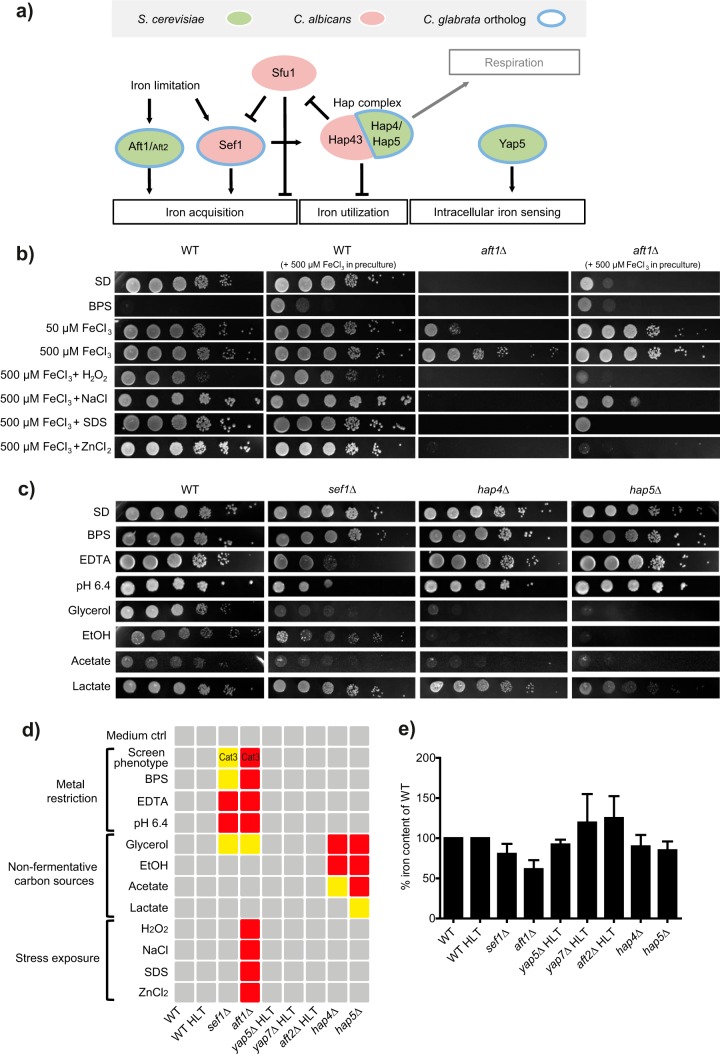
Iron regulators Aft1 and Sef1 are needed for *C. glabrata* growth under iron restriction conditions. (a) Scheme of the distinct iron regulation networks (black connections) in *C. albicans* (red) and *S. cerevisiae* (green) and the orthologs present in the *C. glabrata* genome (circled in blue). (b) Growth of the *aft1*Δ mutant on solid media (SD) under conditions of iron restriction (BPS) and a combination of iron supplementation in the preculture (500 µM FeCl_3_) and in the plate (50 µM FeCl_3_ or 500 µM FeCl_3_) and with oxidative stress (H_2_O_2_), osmotic stress (NaCl), cell membrane stress (SDS), and high metal stress (ZnCl_2_). (c) Growth of regulator deletion strains on solid media (SD) in the presence of iron-associated stressors (BPS, EDTA [pH 6.4]) and on nonfermentative carbon sources (glycerol, EtOH, acetate, lactate). (d) Growth levels of all regulator deletion strains are displayed schematically for all tested stressors as follows: no growth defect, gray box; moderate growth defect, yellow box; severe growth defect, red box. Cat3, category 3 iron screen phenotype; ctrl, control. (e) Intracellular iron content of *C. glabrata* regulator deletion strains of YPD-grown log-phase cells shown as a percentage of the content determined for the corresponding WT strain ± SEM. An unpaired two-tailed *t* test was performed.

The identified regulators Aft1 and Sef1 are known key components in independent and distinct iron regulation networks of the baker’s yeast species *S. cerevisiae* and the pathogen *C. albicans*, respectively. Since these findings suggest overlapping contributions, we decided to analyze both networks in more detail. Therefore, we screened the *C. glabrata* genome *in silico* for potential orthologs of the known iron-associated regulators in *C. albicans* and *S. cerevisiae* and identified seven candidates. These comprised mostly orthologs from the *S. cerevisiae* iron network, including Aft1, its functionally redundant paralog Aft2 (32), and the fungus-specific bZIP transcription factor Yap5, an intracellular iron sensor (33). *C. glabrata* Yap5, along with Yap7, has recently been shown to regulate iron sulfur cluster biogenesis and heme biosynthesis in a global chromatin immunoprecipitation (ChIP) and transcriptome analysis approach (34). Additionally, members of the Hap complex were tested, e.g., Hap4 and Hap5, known to contribute to Hap43-regulated iron homeostasis in *C. albicans* (35) but to respiratory gene expression in *S. cerevisiae* (36) and with an unclear role in *C. glabrata*. Sef1 was chosen as the only direct ortholog from the *C. albicans* iron regulation network present in *C. glabrata*; a *SFU1* ortholog was not found. Figure 2a summarizes these species-specific iron regulatory networks and the *C. glabrata* orthologs. These candidate regulator knockout strains were included in the growth screen along with *aft1*Δ and *sef1*Δ mutants but did not show any growth defects under low-iron conditions (see Dataset S1 in the supplemental material). In contrast, the *aft1*Δ mutant showed a severe iron-dependent growth defect and also showed increased sensitivity to oxidative, osmotic, high metal, and cell membrane stress (Fig. 2b). Growth under various metal restriction conditions, such as the presence of the general metal chelator ethylenediaminetetraacetic acid (EDTA) or of the iron chelator bathophenanthrolinedisulfonic acid (BPS), or under alkaline pH conditions, which reduce iron solubility, revealed a moderate defect for the *sef1*Δ mutant but not for the *hap4*Δ, *hap5*Δ, *yap5*Δ, *yap7*Δ, or *aft2*Δ mutant (Fig. 2c and d). Instead, growth of the *hap4*Δ and *hap5*Δ mutants was impaired on the nonfermentative carbon sources glycerol, lactate, ethanol, and acetate, which require respiration for energy generation, indicating a role for these genes in respiration similar to that in *S. cerevisiae*. Finally, we determined the intracellular iron content for all *C. glabrata* regulator knockout strains and found decreased iron content only in the *aft1*Δ and *sef1*Δ strains (Fig. 2e). Taking the data together, the *C. glabrata* phenotypes seen upon deletion of *AFT1*, *HAP4*, or *HAP5* resemble the *S. cerevisiae* phenotypes, while the *SEF1* phenotype is more reminiscent, at least in part, of those of *C. albicans* and other pathogenic fungi.

### The iron-dependent phenotype of Sef1 is specific to *C. glabrata* and not present in *S. cerevisiae.*

**FIG 3:**
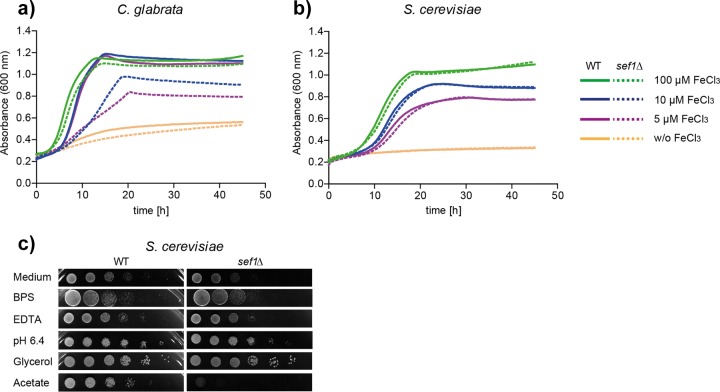
Sef1 performs an iron-related role in *C. glabrata* but not in *S. cerevisiae*. (a) Growth of the *C. glabrata* WT and *sef1*Δ strains under conditions of iron restriction (SD+200 µM BPS) and iron supplementation (5 µM, 10 µM, or 100 µM FeCl_3_). The generation times (means ± standard deviations) of the WT and *sef1*Δ strains differed significantly in the presence of 5 µM FeCl_3_ (4.8 ± 1.14 h versus 13.2 ± 3.79 h; *P* < 0.05) and 10 µM FeCl_3_ (3.7 ± 0.35 h versus 9.6 ± 1.67 h; *P* < 0.01) but not in the presence of 100 µM FeCl_3_ (2.8 ± 0.09 h versus 3.1 ± 0.22 h). Data from a representative replicate are displayed (*n* = 3). (b) Growth levels of *S. cerevisiae* WT and *sef1*Δ as described for panel a showed no significant differences. A representative replicate is displayed. *n* = 3. (c) Growth of *S. cerevisiae* WT and *sef1*Δ in the presence of the stressors BPS, EDTA (pH 6.4), glycerol, and acetate.

Sef1 is known to play a fundamental role in the *C. albicans* iron regulation network. Our findings likewise suggest an—albeit less pronounced—iron-related role in *C. glabrata*. The function of Sef1 in *S. cerevisiae* is thus far unknown, and so we investigated a possible connection to iron homeostasis similar to that seen with *C. glabrata* and *C. albicans*. Growth curve analysis showed growth representative of a defect under low-iron conditions (5 µM and 10 µM FeCl_3_) for a *C. glabrata sef1*Δ mutant which was restored to wild-type (WT) levels by addition of 100 µM FeCl_3_ (Fig. 3a). In contrast, the *S. cerevisiae sef1*Δ mutant results were indistinguishable from the WT results under all conditions (Fig. 3b). Similarly, exposure to growth conditions that included the presence of iron-limiting stressors such as BPS, EDTA, or alkaline, as well as of glycerol as a sole carbon source, caused no growth defects of *S. cerevisiae sef1*Δ (Fig. 3c), in clear contrast to *C. glabrata sef1*Δ (Fig. 2c). Instead, *S. cerevisiae sef1*Δ, but not *C. glabrata sef1*Δ, was strongly attenuated in growth on the nonfermentative carbon source acetate. Taken together, these data suggest a species-specific role for Sef1 which in *C. glabrata* resembles the function of Sef1 in *C. albicans* more than in *S. cerevisiae.*

### Aft1 and Sef1 have different regulatory functions under iron starvation conditions.

**FIG 4:**
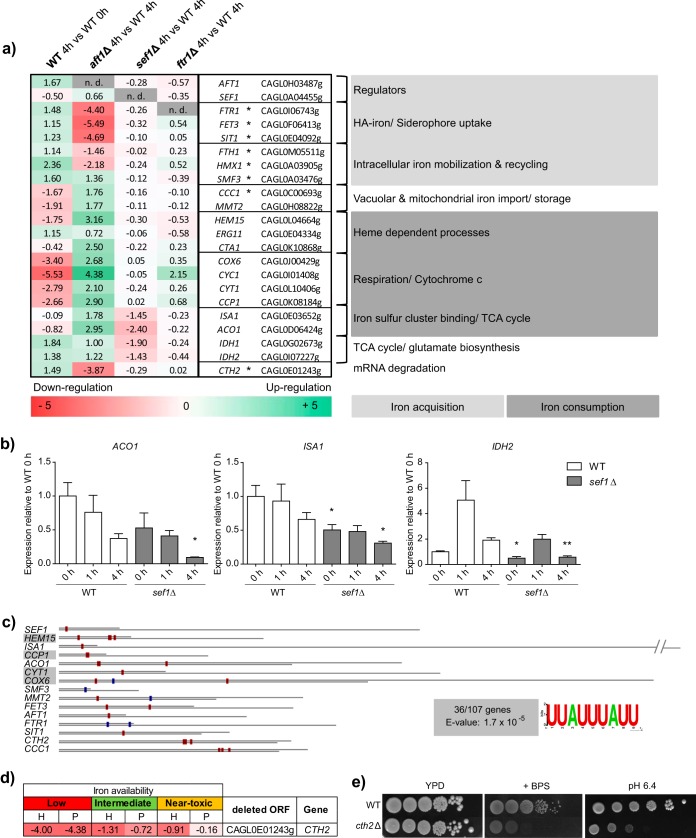
Comparative transcriptional profiling shows major dysregulation of iron metabolism-associated genes in the *aft1*Δ mutant and reveals that Cth2 is a potential posttranscriptional regulator. (a) Relative mRNA levels of iron-associated genes in the WT strain compared to the *aft1*Δ, *sef1*Δ, and *ftr1*Δ mutants under iron starvation conditions. Microarray data are displayed in a normalized log_2_ scale for downregulation (negative numbers, shaded red), upregulation (positive numbers, shaded green), or no detected expression (n.d.) of target genes. Asterisks (*) indicate target genes that carry one or multiple copies of the Aft1 consensus-binding site PyPuCACCCPu in the 5′ UTR. (b) qRT-PCR expression of the following genes affected by *SEF1* deletion relative to the levels seen with the WT at 0 h: *ACO1* (encoding mitochondrial aconitase), *ISA1* (encoding an iron sulfur cluster assembly), and *IDH2* (encoding isocitrate dehydrogenase). Data represent means of results of biological triplicate experiments ± SEM (nd, no expression detected). Data are from an unpaired Student’s *t* test and represent results of comparisons to WT results from the same time point (*, *P* value < 0.05; **, *P* value < 0.01). (c) The postulated target consensus motif of the Cth2 mRNA-degrading protein (5′-UUAUUUAUU-3′) is found in the downstream sequences of 36 (of 106) genes normally downregulated in the WT strain upon iron starvation (0 h to 4 h) (volcano plot; 2-fold downregulation; *P* value = 0.05; false-discovery-rate [FDR] corrected) but is artificially upregulated in the *aft1*Δ strain (4 h against WT) (volcano plot; 2-fold upregulation; *P* value = 0.05; FDR corrected). Motifs (red box, forward strand; blue box, reverse strand) are shown for the previously displayed striking microarray genes in the annotated 3′ intergenic region (lower gray line) or the predicted 3′ UTR of the target gene (upper gray line) in proportion to the actual length (1 dot represents 4 bp; // indicates a discontinued display). (d) Growth of the *cth2*Δ mutant under low-iron conditions (10 µM FeCl_3_), intermediate-iron conditions (500 µM FeCl_3_), and near-toxic iron conditions (40 mM FeCl_3_) in Chelex-treated SD (pH 5.8) compared to the WT strain (zero, shaded white) determined by assessing the time of half-maximal growth (H) and the plateau height (P). Positive numbers indicate improved growth (shaded green), and negative numbers indicate growth defects (shaded red). Data represent means of results from triplicate experiments. (e) Growth of the *cth2*Δ mutant in the presence of the iron chelator BPS (50 µM) and under alkaline conditions (pH 6.4) on solid media.

Our results indicate roles for both Aft1 and Sef1 in the regulation of iron homeostasis in *C. glabrata*. To gain insight into the target genes of these transcription factors, we compared the transcription profiles of the wild-type, *aft1*Δ, and *sef1*Δ strains under iron-restricted conditions. An *ftr1*Δ strain, deficient for the main high-affinity iron transporter, was included as a control. At 4 h after a shift from 5 µM FeCl_3_ to iron-free medium, genes involved in iron uptake processes (*FTR1*, *FET3*, *SIT1*) and iron recycling processes (*FTH1*, *HMX1*, *SMF3*) were strongly upregulated in the WT (Fig. 4a). However, these genes were significantly less transcribed in the *aft1*Δ mutant than in the WT after 4 h. A consensus-binding motif for Aft1 (PyPuCACCCPu) in *S. cerevisiae* is known (21). We found many genes associated with iron uptake or recycling containing one or more copies of this binding motif in their promoter regions (marked with an asterisk [*] in Fig. 4a). The data in Dataset S2 in the supplemental material indicate all *C. glabrata* ORFs (open reading frames) with at least one detectable upstream Aft1-binding motif as possible regulation targets. Remarkably, there was a large set of genes involved in iron-consuming processes (e.g., *CYC1*, *COX6*, *CCP1*, *CCC1*, *HEM15*), and those genes were strongly downregulated in the WT cells upon iron starvation but not in *aft1*Δ cells. Despite its phenotypic effects on growth and iron content, deletion of *SEF1* had only subtle effects on the transcription profile under iron deprivation conditions. Genes involved in the TCA cycle and in glutamate biosynthesis in a broader sense (*ACO1*, *IDH1*, *IDH2*), as well as genes with iron sulfur cluster-dependent functions (*ACO1*, *ISA1*), were transcribed to a lesser degree under iron starvation conditions upon *SEF1* deletion. Transcriptional downregulation was verified by quantitative reverse transcription-PCR (qRT-PCR) for the aconitase gene *ACO1*, the isocitrate dehydrogenase gene *IDH2*, and the gene encoding the iron sulfur cluster protein Isa1*.* These data indicate a potential role for Sef1 in TCA cycle regulation (Fig. 4b). Under replete-iron conditions, interestingly, some genes coding for iron-requiring enzymes (*CCP1*, *CYT1*, *HEM15*, *CCC1*, *ACO1*) showed a tendency of increased transcription in the *sef1*Δ mutant compared to the WT (see Dataset S3c in the supplemental material), pointing to a possible role of Sef1 in the “fine-tuning” of iron consumption processes. In summary, our data suggest a dual role of Aft1: as the major transcriptional activator of iron uptake and recycling processes and as a repressor of iron consumption processes. Additionally, Sef1 seems to regulate specific iron-associated processes such as the TCA cycle under iron restriction conditions.

### *CTH2* is induced under iron starvation conditions via Aft1 to downregulate iron consumption and *SEF1.*

**FIG 5:**
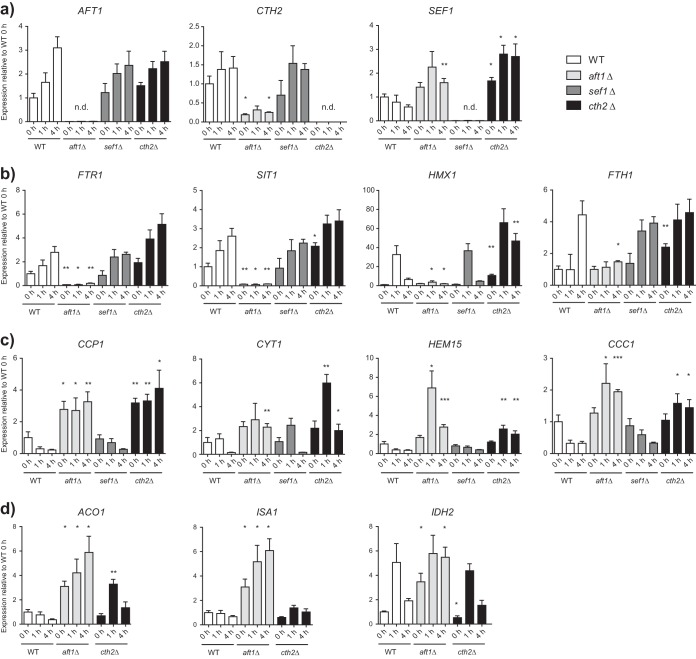
Expression analysis of iron-associated target genes by qRT-PCR. Data represent gene expression of target genes for the WT, *aft1*Δ, *cth2*Δ, and *sef1*Δ strains at 0 h, 1 h, and 4 h. After 4 h of preincubation under low-iron conditions (5 µM FeCl_3_), the 0-h sample was obtained. Residual cells were then incubated without iron for 1 h or 4 h. Target gene expression was normalized to that of housekeeping genes *EFB1* and *EFT2*. Expression is shown relative to that of the WT at 0 h in biological triplicate experiments ± SEM (n.d., no expression detected). Data are from an unpaired Student’s *t* test and represent results of comparisons to WT results from the same time point (*, *P* < 0.05; **, *P* < 0.01; ***, *P* < 0.005). (a) Expression of the genes encoding regulators of iron metabolism: *AFT1* and *SEF1* (encoding transcription factors) and *CTH2* (encoding an mRNA-degrading protein)*.* (b) Expression of the iron acquisition genes *FTR1* (encoding an HA-iron transporter), *SIT1* (encoding a siderophore transporter), *HMX1* (degrading heme oxidase), and *FTH1* (encoding a vacuolar HA-iron exporter) under iron deprivation conditions. (c) Expression of the iron consumption genes *CCP1* (encoding mitochondrial cytochrome *c* peroxidase), *CYT1* (encoding cytochrome *c*), *HEM15* (encoding heme synthesis), and *CCC1* (encoding a vacuolar HA-iron importer) under iron deprivation conditions. (d) Expression of the following genes affected by *SEF1* deletion: *ACO1* (encoding mitochondrial aconitase), *ISA1* (encoding an iron sulfur cluster assembly), and *IDH2* (encoding isocitrate dehydrogenase).

According to our transcription data, iron-consuming processes were downregulated in the WT but not in the *aft1*Δ mutant during iron starvation (Fig. 4a). To investigate the underlying mechanism, we therefore selected genes downregulated in the WT at 4 h of starvation but with at least 2-fold higher expression in the *aft1*Δ mutant (*aft1*Δ mutant at 4 h versus the WT at 4 h). These 106 ORFs were analyzed for a common motif in their flanking regions using the bioinformatics tool FIMO (37). We found the motif 5′-TTATTTATT-3′ to be located in the 3′ untranscribed region (UTR) of 36 of these ORFs (*E* value 1.7 × 10^−5^) in single or multiple copies. A search for this motif in the intergenic regions of all known *C. glabrata* ORFs (see Dataset S4 in the supplemental material) detected additional iron metabolism-associated genes such as *AFT1*, *SEF1*, *ACO1*, and *CCC1* (Fig. 4c). In fact, most of these genes were then found to be deregulated in the *aft1*Δ mutant and were originally excluded only because of our stringent threshold. Interestingly, this motif is known in *S. cerevisiae* as the nonamer consensus recognition motif of mRNA-degrading protein Cth2 (also known as Tis11). *S. cerevisiae* Cth2 (ScCth2), along with its paralog Cth1, was found specifically to target mRNA of iron-dependent genes for degradation via binding to this motif (22, 24), whereas the corresponding Cth2 protein in *C. glabrata* was still uncharacterized. Interestingly, *C. glabrata* has no ortholog of *CTH1*. To elaborate the role of Cth2 in *C. glabrata*, we constructed deletion mutants and performed growth assays. Growth of a mutant lacking *CTH2* was strongly impaired under low-iron conditions in liquid culture (Fig. 4d), and the mutant showed susceptibility to BPS and alkaline growth conditions (Fig. 4e). In agreement with this, *CTH2* transcription was highly upregulated upon iron starvation in the WT but not in the *aft1*Δ mutant (Fig. 4a). Since the *CTH2* promoter also carries the Aft1 recognition motif, we postulated *CTH2* to be a target gene of Aft1. In fact, qRT-PCR showed that lack of *AFT1* abolished expression of *CTH2* (Fig. 5a). Furthermore, expression of *SEF1* was elevated in both *aft1*Δ and *cth2*Δ strains of *C. glabrata*. *In silico* analysis revealed that the *S. cerevisiae* ortholog of *SEF1*, in contrast, lacks the nonamer-binding site for Cth2 (2.5 kb up- and downstream of the ORF), reflecting its lack of iron-related function in this fungus. Taking the results together, we have identified *C. glabrata* Cth2 as a potential Aft1-induced mediator of mRNA degradation of iron-associated genes, a group which includes *SEF1*.

### Cth2 is required for limiting iron consumption and plays a minor role in iron uptake regulation.

Microarray transcriptional profiling analysis of the *aft1*Δ mutant had indicated a lack of induction of iron acquisition genes under iron deprivation conditions, which we confirmed by qRT-PCR for *FTR1*, *SIT1*, *HMX1*, and *FTH1* (Fig. 5b). As the mRNA levels of all of these genes were also consistently elevated in the *cth2*Δ mutant, Cth2 seems to exert its posttranscriptional mRNA degradation on mRNA transcripts of highly expressed iron acquisition genes under iron starvation conditions. Consistently, lack of *CTH2* did not influence their already low expression levels under iron-replete conditions (see Dataset S3b in the supplemental material). We similarly analyzed expression levels of genes associated with iron consumption (Fig. 5c), including respiration-associated genes such as *CCP1* (cytochrome *c* peroxidase) and *CYT1* (heme-dependent electron transport), *HEM15* (heme synthesis), and *CCC1* (vacuolar iron import). Consistent with our previous results, the mRNA levels of these genes were downregulated upon iron deprivation in the WT, but transcript levels were much higher in the *aft1*Δ mutant. Similarly, lack of *CTH2* resulted in a significant and comparable increase in mRNA levels. Under iron-replete conditions, the iron consumption processes were mainly upregulated in the WT. Lack of *CTH2*, on the other hand, either slightly elevated the levels of respiration-associated genes or decreased them for other iron consumption processes (see Dataset S3c). Taking the results together, Cth2 is strictly needed for downregulating iron consumption processes under iron restriction conditions. Additionally, our data point to a continuous degradation of highly transcribed mRNAs involved in iron uptake processes.

### C. *glabrata* mutants lacking *AFT1* and *SEF1* display diminished survival in human blood.

**FIG 6:**
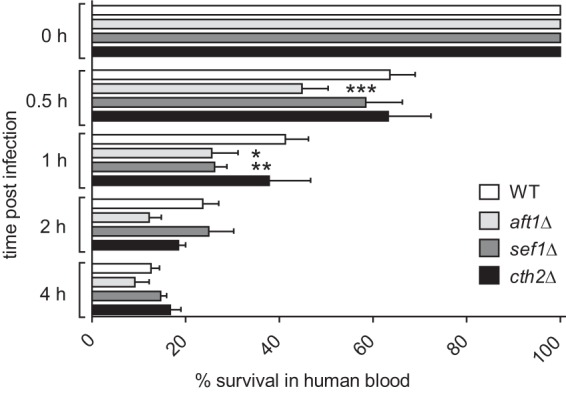
Aft1 and Sef1 are needed for survival in human blood. Human blood was infected with the *C. glabrata* WT, *aft1*Δ, *sef1*Δ, and *cth2*Δ strains, and survival at 37°C was determined by CFU plating after the indicated time points (0 h, 0.5 h, 1 h, 2 h, and 4 h). Data are shown as percent survival of the WT at 0 h (means ± standard deviation). Data represent results of a paired one-way analysis of variance (ANOVA) of comparisons to WT results from the same time point (*, *P* < 0.05; **, *P* < 0.01; ***, *P* < 0.001).

We have shown that Aft1, Cth2, and Sef1 are important for the regulation of iron metabolism *in vitro*. To elaborate whether these factors also play a role in the host, we analyzed the survival of *C. glabrata aft1*Δ, *cth2*Δ, and *sef1*Δ strains in an *ex vivo* human blood model mimicking, to some extent, the dissemination phase of systemic fungal infections (Fig. 6). There, the *aft1*Δ mutant showed a clear and significant reduction in early survival in blood. Survival of the *sef1*Δ mutant was likewise significantly attenuated early during exposure to blood (at 1 h and with the tendency already seen at 0.5 h), whereas, remarkably, deletion of *CTH2* did not affect survival at any time point. A reduction of *aft1*Δ survival was observable also at later time points, albeit without reaching the statistical significance levels of the earlier samples. Hence, both Aft1 and Sef1 seem to be required for full survival in blood, especially during the early interaction. Assuming iron-scarce conditions during the initial immune defense, this would be in good agreement with the proposed roles of both transcription factors in iron homeostasis of *C. glabrata*.

## DISCUSSION

Iron homeostasis networks have been studied in a variety of pathogenic fungi, and they are known to play important roles in virulence (38). Although transcriptomics data have been obtained during changes in environmental iron levels for *C. glabrata* (39), our knowledge of genes which are essential for iron homeostasis, of their integration into the complex regulatory networks, and of their role in virulence remained limited. We therefore employed a screen of deletion mutants where we mimicked both iron deprivation and iron overload to detect genes involved in iron homeostasis. Using this setup, we identified 100 deletion mutants with relevant growth defects. We were able to confirm the important roles of various iron-associated genes described previously in *C. glabrata*, such as *FTR1*, *FET3*, and *YFH1* (40), or in *C. albicans* and *S. cerevisiae*, such as *ATM1* (29, 31). However, most of our candidate genes have not been associated previously with iron homeostasis in *C. glabrata*. A shortage of iron represents a common challenge in most host niches of *C. glabrata* (41, 42); hence, we considered mutant growth defects under low-iron conditions especially informative for the identification of possible virulence factors. Strikingly, we identified Aft1 as an essential transcription factor of *C. glabrata* necessary for growth under low-iron conditions via activation of iron uptake and intracellular recycling. *aft1*, known as the major transcriptional activator of the iron regulon in *S. cerevisiae* (21, 22, 31), was presumed an essential gene in *C. glabrata* by Srivastava et al. (40), since a knockout strain could not be obtained. Here we show that *AFT1* is not essential, or, more precisely, is conditionally essential, as it is required only under conditions of low to moderate iron concentrations and not under conditions of highly elevated concentrations. Hence, we were able to show that *C. glabrata*, like its closest relative—the baker’s yeast *S. cerevisiae*—uses an Aft1-dominated system for iron homeostasis. To our knowledge, *C. glabrata* is the first known pathogen engaging an Aft1-dependent system as the main iron regulation strategy. Considering that levels of iron availability and sources of iron, as well as the physical conditions (temperature, pH, oxygen levels), differ greatly between the terrestrial environment of *S. cerevisiae* and a pathogen’s animal host niches, it is astonishing that *C. glabrata* utilizes the Aft1-dependent system. Thus, our findings point to a rather unusual regulation strategy for *C. glabrata* compared to other fungal pathogens.

Interestingly, according to our transcription data, *C. glabrata* reacts to iron deprivation in a time-dependent manner and first activates the uptake mechanisms (*FTR1*, *SIT1*), followed by the mobilization of vacuolar storages (*FTH1*), and intermediately and transiently resorts to heme recycling (*HMX1*). These observations indicate a tightly controlled and timed response to iron deprivation, probably depending on the increasing magnitude of intracellular iron deficiency. In addition to the upregulation of iron acquisition genes, we also observed a marked downregulation of genes involved in iron consumption processes.

In *S. cerevisiae*, the Aft1 regulon is known to recruit the mRNA-degrading protein Cth2 (also known as Tis11), which posttranscriptionally reduces the abundance of iron consumption-related mRNAs under conditions of iron deprivation (22, 24). We were able to identify a functional homolog of *CTH2* in *C. glabrata* which is also positively regulated by Aft1. As in *S. cerevisiae*, we found the highly conserved motif 5′-UUAUUUAUU-3′ in the 3′ UTR of target mRNAs of Cth2. In both species, these are transcripts coding especially for proteins with a role in iron consumption (24). Additionally, we have also observed a moderate effect of Cth2 on the mRNA levels of iron acquisition genes in *C. glabrata*. In contrast to *S. cerevisiae* (24), the Cth2-binding motif is present in *FTR1*, *FET3*, and *SIT1* transcripts, pointing to a novel role in posttranscriptional fine-tuning of iron uptake likely via an increased turn-over of mRNA, which could be advantageous for survival in niches with often rapidly shifting iron levels.

Notably, Cth2 has a partially redundant paralog, Cth1, in *S. cerevisiae*. Although there, both *CTH1* and *CTH2* are activated by Aft1 under iron deprivation conditions, *CTH1* is expressed only transiently early during iron deficiency, while *CTH2* expression persists during prolonged iron limitation conditions. Additionally, autoregulation and *trans*-regulation have been proposed for those two mRNA degradation mediators, since both carry a 5′-UUAUUUAUU-3′ recognition motif (24, 43, 44). *C. glabrata* is lacking a second copy of *CTH2*, indicating that while it seems to rely on the Cth system for downregulation, its specific implementation differs from that used by *S. cerevisiae*. Since Cth2 carries its own recognition motif, an autoregulatory loop seems likely. Remarkably, *C. albicans* also possesses a single Cth2 ortholog called Zfs1. However, Zfs1 target transcripts are not related to iron homeostasis but rather to biofilm formation (45). The only other fungus with a functionally described Cth2 ortholog, *Schizosaccharomyces pombe*, also displays no transcriptional alterations of its iron homeostasis genes when this gene is deleted (46, 47). These observations indicate a mode of recognition by Cth2 that is evolutionarily conserved between *C. glabrata* and *S. cerevisiae*, with a large set of overlapping target genes (24), while the sets of regulated target genes differ greatly between all other investigated species. To our knowledge, *C. glabrata* is thus the first example of a pathogenic fungus using its Cth2 ortholog to downregulate iron homeostasis-associated transcripts. Further factors may play a role in this iron-level-dependent transcript degradation process, as most but not all decreases in the mRNA level were dependent on the presence of Cth2.

In contrast, other pathogenic fungi such as *Aspergillus fumigatus*, *Cryptococcus neoformans*, and *C. albicans* generally rely on iron-dependent regulation via GATA factor orthologs and the CCAAT-binding complex (12, 48–51). Although this system is broadly conserved among ascomycetes and partly in basidiomycetes, there are a few species-specific modifications—for instance, the incorporation of Sef1 orthologs into this network (49). In *C. albicans*, Sef1 is a pivotal activator of iron uptake genes and thus plays an essential role during iron deficiency and for virulence in mice (19). In this study, we showed that, surprisingly, the *C. glabrata* Sef1 ortholog has an iron-related function although the fungus is not using the GATA factor and CCAAT-binding complex for iron-dependent regulation. A Sef1 ortholog can also be found in *S. cerevisiae*, but, in contrast to *C. glabrata*, we have found no connection of Sef1 to iron metabolism in baker’s yeast. These findings strongly suggest a novel hybrid iron regulation system for *C. glabrata* which combines features from the networks of *S. cerevisiae* (Aft1) and *C. albicans* (Sef1). The *Candida* and *Saccharomycetaceae* clade diverged evolutionarily from each other, with the latter partly undergoing a whole-genome duplication (WGD) event (52). During evolution, a complete rewiring of the iron regulation network from the likely more ancient GATA/CCAAT-binding complex system to Aft1/Cth2 must have evolved in the *Saccharomycetaceae* branch, accompanied by the loss of the GATA factor and the iron-associated CCAAT-binding factor function. Although the components of the CCAAT-binding complex (e.g., Hap4, Hap5) are still present in *S. cerevisiae*, they are now involved in the regulation of aerobic respiration (36). *C. glabrata*, as part of the post-WGD *Saccharomycetaceae* clade (53), underwent the same process but apparently has maintained a residual Sef1 function, resulting in a hybrid form of iron regulation with major features derived from the *S. cerevisiae* system and minor features retained from the system used by *C. albicans* and other pathogenic fungi.

The full role of Sef1 in the *C. glabrata* iron network still eludes us; however, we found important functional characteristics of Sef1 during iron starvation. Growth defects of the *sef1*Δ mutant under conditions of iron deprivation and of alkaline pH (with its concomitant low iron solubility), as well as the *in silico* analysis, place it firmly in the *C. glabrata* iron homeostasis regulatory network. Surprisingly, despite its phenotype, the transcriptome of the *sef1*Δ mutant did not differ strongly from that of the WT. This is especially striking in comparison to the large effect that *SEF1* deletion has on *C. albicans* iron-dependent transcriptional responses, where Sef1 is a major activator of iron uptake-related genes (reference 49 and own unpublished observations). In the process of the change to an Aft-dependent system, most of these interactions seem to have been lost within the *Saccharomycetaceae* branch. However, at least in *C. glabrata*, very specific changes in gene expression were observed for genes associated with the (iron-requiring) TCA cycle (*ACO1*, *IDH1*, *IDH2*) as well as for genes encoding known iron sulfur cluster-containing proteins (*ACO1*, *ISA1*).

Interestingly, mammalian Aco1 has dual functions that depend on iron availability. Under conditions of low iron availability—when it is known as IRP1—it acts as an RNA-binding protein and inhibits translation initiation of mRNAs related to iron-consuming and -storing processes, such as ferritin mRNA (54, 55). On the other hand, IRP1 binding can also stabilize target RNAs under iron limitation conditions, e.g., the mRNA coding for the transferrin receptor, preventing degradation to enhance the transferrin-mediated iron uptake pathway (56). However, under high-iron conditions, IRP1 acquires an active iron sulfur cluster and is converted to a fully functional cytosolic aconitase (57–59). Although plants and fungi seem to lack this iron regulatory switch in function for Irp1/Aco1 (60, 61), the mitochondrial aconitase remains a crucial iron-dependent protein of the TCA cycle in fungi (29, 62).

Furthermore, Isa proteins have been proposed to function in the maturation of aconitase-like iron sulfur cluster proteins in *S. cerevisiae* (63, 64). Since we observed less expression of Isa1 and Aco1 in the *sef1*Δ mutant, the two proteins might be part of the same process, suggesting a need for Sef1 for the assembly of specific iron sulfur cluster proteins, such as Aco1 via Isa1. In *S. cerevisiae*, various iron sulfur proteins have been implicated in iron sensing, where the status of iron loading indicates the cellular lack or presence of iron (63, 65, 66). Since Sef1 is needed to sustain basal expression of *ISA1* and *ACO1* according to our data, it might be necessary to ensure the presence of specific iron sulfur cluster proteins under iron starvation conditions, which may then act as iron sensors, signaling iron deprivation to the cell by lack of iron binding. Overall, it seems that Sef1 has a very specialized function in the hybrid iron homeostasis regulation of *C. glabrata*, with the Aft1/Cth2 system bearing the brunt of transcriptional regulation under iron starvation conditions and Sef1 acting on specific pathways.

We were also interested in the role of Aft1 and Sef1 under conditions resembling a human infection. Establishment of systemic disease caused by *Candida* spp. requires (i) entry into the bloodstream by overcoming epithelial barriers or by colonizing of intravenous catheters (67) and (ii) initial dissemination via the bloodstream. These processes frequently lead to sepsis with significant associated morbidity and mortality and to high health care-associated costs (68, 69). To survive in the blood and successfully disseminate, *Candida* spp. evidently have to undergo drastic changes of metabolism. Metal availability is especially tightly restricted in blood to facilitate nutritional immunity. Free iron availability is very low (10^−24^ M Fe^3+^) (19) and below the minimal concentration required for microbial growth (10^−6^ to 10^−7^ M Fe^3+^) (70). Especially for *C. glabrata*, which (in contrast to *C. albicans*) is not known to use hemoglobin or transferrin for iron acquisition (8, 15), survival in this environment is likely dependent on tight and precise regulation of the balance between iron uptake and consumption. In *C. albicans*, Sef1 is crucial for iron uptake in bloodstream infections, as shown by severely decreased virulence and recovery from murine organs of a *sef1*Δ/Δ mutant (49). Here, we have shown that Sef1 is a transcriptional regulator required for early survival in human blood in *C. glabrata* as well, while an even more pronounced decrease in survival was observed for the *aft1*Δ (main regulator) mutant. We found that both genes are thus needed for early survival in blood, while at later time points, killing by immune cells probably reaches a level which starts to occlude the iron-dependent effects. It is noteworthy, however, that during dissemination, fungi are likely exposed only briefly to the bloodstream before they enter the target organs and hence that early survival may be crucial for successful systemic infections (71). These data hence underline the prominent role of Aft1 and Sef1 in *C. glabrata* iron homeostasis and suggest a role of these factors in human virulence.

**FIG 7:**
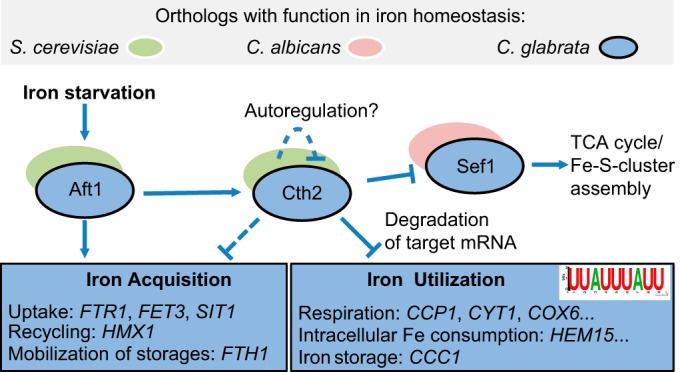
Model of iron metabolism regulation in *C. glabrata*. In *C. glabrata* (blue), similarly to *S. cerevisiae* (green), Aft1 acts as an activator of iron acquisition genes under iron starvation conditions (*FTR1*, *FET3*, *SIT1*), while it also induces expression of *CTH2*. Cth2, a mediator of posttranscriptional mRNA degradation, targets transcripts carrying the recognition motif 5′-UUAUUUAUU-3′, predominantly associated with iron consumption processes (*HEM15*, *CCP1*, *ACO1*). Additional postulated functions of Cth2 (dashed lines) also include the negative regulation of iron acquisition in a moderate manner and an autoregulatory role. The transcription factor *SEF1*, which is important in the *C. albicans* iron regulon (red), is also a Cth2 target gene in *C. glabrata* which has a specific impact on the TCA cycle and on iron sulfur cluster (Fe-S cluster) assembly.

To summarize, we propose a model for the regulation of iron metabolism in *C. glabrata* under conditions of iron deprivation (Fig. 7). Aft1 acts as the major transcriptional activator of iron acquisition processes and of *CTH2* expression. Subsequently, Cth2 mediates posttranscriptional degradation of specific transcripts carrying a conserved recognition motif. Potential targets include predominantly iron consumption-related genes and *SEF1*, which itself regulates a subset of genes. Remarkably, this kind of iron regulation system has not been described in any other fungus and seems to be unique to the opportunistic pathogen *C. glabrata*. It mostly resembles the regulation system of the baker’s yeast *S. cerevisiae*, whereas some components from *C. albicans* are retained. These network adaptions might be of advantage for virulence of *C. glabrata*, contributing to its successful life as a pathogen despite its close evolutionary relations to *S. cerevisiae*.

## MATERIALS AND METHODS

### Strains.

The mutants screened in this study comprised the strains described in Schwarzmüller et al. (25) as well as *C. glabrata* gene knockout strains generated in this study, constructed in the ATCC 2001 wild-type strain using the same gene deletion strategy. The latter included orthologs of *S. cerevisiae* or *C. albicans* high- and low-affinity iron uptake systems (*fet3*Δ, *ftr1*Δ, and *fet4*Δ mutants) and transcriptional regulators (*aft1*Δ, *sef1*Δ, *hap4*Δ, and *hap5*Δ mutants) (Table 1). Briefly, the gene of interest (GOI) was replaced via homologous recombination by a cassette containing constant flanking regions (U1 and D1), specific up- and downstream bar codes, and the nourseothricin resistance marker *NAT1*. Cassettes were fused with 500-bp to 1,000-bp target gene-specific 5′ and 3′ flanks and integrated into XbaI-linearized pUC19 vector using an Infusion HD cloning kit (Clontech). The insertion was PCR amplified (with primers GOI fwd and GOI rev) and used for *C. glabrata* transformation by a modified heat shock method (72) (with 45°C heat shock for 15 min). The transformants were plated onto yeast extract-peptone-dextrose (YPD; 1% yeast extract, 2% Bacto peptone, 2% glucose) agar containing 250 µg/ml nourseothricin, supplemented where necessary with 500 µM FeCl_3_ (strains *ftr1*Δ and *aft1*Δ). Knockout strains were verified by sequencing and Southern blotting (*NAT1*-specific probe; primers NAT fwd South and Nat rev South). All primers are listed in Dataset S5 in the supplemental material.

**TABLE 1:** Strains used in this study^a^

Strain name	Description	Source or reference
*C. glabrata*		
WT	*C. glabrata* WT strain ATCC 2001	American Type Culture Collection
WT HLT	*C. glabrata* WT strain for collection ATCC 2001; *his3*Δ::FRT *leu2*Δ::FRT *trp1*Δ::FRT	25
Deletion mutant collection (GOIΔ HLT)	ATCC 2001; *goi*δ::*NAT1 his3*Δ::FRT *leu2*Δ::FRT *trp1*Δ::FRT	25
*aft1*Δ	ATCC 2001; *CAGL0H03487g*Δ::*NAT1*	This study
*ftr1*Δ	ATCC 2001; *CAGL0I06743g*Δ::*NAT1*	This study
*sef1*Δ	ATCC 2001; *CAGL0A04455g*Δ::*NAT1*	This study
*fet3*Δ	ATCC 2001; *CAGL0F06413g*Δ::*NAT1*	This study
*fet4*Δ	ATCC 2001; *CAGL0F00187g*Δ::*NAT1*	This study
*cth2*Δ	ATCC 2001; *CAGL0E01243g*Δ::*NAT1*	This study
*hap4*Δ	ATCC 2001; *CAGL0K08624g*Δ::*NAT1*	This study
*hap5*Δ	ATCC 2001; *CAGL0K09900g*Δ::*NAT1*	This study
*S. cerevisiae*		
BY4741	*S. cerevisiae* WT strain; MAT a; *his3*Δ 1; *leu2*Δ 0; *met15*Δ 0; *ura3*Δ 0	EUROSCARF^b^
*YBL066c*Δ*/ sef1*Δ	BY4741; Mat a; *his3*Δ 1; *leu2*Δ 0; *met15*Δ 0; *ura3*Δ 0; *YBL066c*::kanMX4	EUROSCARF^b^

a GOI (gene of interest); HLT (auxotrophy for histidine, leucine, and tryptophan); WT (wild type).

b http://www.euroscarf.de.

### Screen for iron-dependent growth defects.

*C. glabrata* cultures were grown in 96-well plates (TPP) at 37°C and 180 rpm in three consecutive overnight cultures by 1:50 transfers: first, in rich medium (YPD); second, in synthetic defined medium (SD; 0.67% yeast nitrogen base [YNB], 2% glucose, 0.079% complete supplement mix [CSM; Formedium]); and third, in citrate-buffered SD (pH 5.8) supplemented with 200 µM bathophenanthrolinedisulfonic acid (BPS). The optical density at 600 nm (OD_600_) of the last culture was adjusted to 0.1 in iron-free water for growth in iron-free citrate-buffered SD (pH 5.8). This medium was pretreated overnight with Chelex 100 (Sigma) to remove metals, the Chelex 100 beads were removed by filtration, and noniron metals were restored (100 mg/ml CaCl_2_, 500 mg/ml MgSO_4 ⋅_ 7H_2_O, 400 µg/ml MnSO_4_, 40 µg/ml CuSO_4_, 400 µg/ml ZnSO_4 ⋅_ 7H_2_O). Defined amounts of FeCl_3_ were added to obtain low-iron conditions (10 µM FeCl_3_), intermediate iron conditions (500 µM FeCl_3_), or near-toxic iron conditions (40 mM FeCl_3_). Then, 20 µl of the *C. glabrata* inoculum was added to 180 µl of each test medium. Growth was followed by determination of the OD_600_ at 37°C in a Tecan Infinite 200 enzyme-linked immunosorbent assay (ELISA) reader (with intermittent shaking) for 2 days. The relative growth levels of the mutants were determined by the time of half-maximal OD_600_ (H) and the final plateau height of the stationary phase (P) relative to those determined for the wild type. The values were normalized to the wild-type control values, and the log_2_ of this ratio was calculated; hence, negative numbers indicate growth defects. The screen was performed once for all 648 mutants and repeated twice to obtain triplicate measures whenever a possible phenotype was seen.

### Serial dilution growth tests.

Precultures were prepared in SD supplemented with CSM and optionally with 500 µM FeCl_3_. Sensitivity of *C. glabrata* strains to stressors was tested by spotting 1:10 serial dilutions on agar containing H_2_O_2_ (SD, 12 mM), NaCl (SD, 1.1 M), sodium dodecyl sulfate (SDS) (SD, 60 µg/ml), ZnCl_2_ (SD, 12 mM), BPS (YPD, 50 µM), or EDTA (SD, 8 µM). The alternative carbon sources used instead of glucose were glycerol (3%), sodium lactate (2%), ethanol (2%), and sodium acetate (4%). Growth at a more alkaline pH was tested by buffering SD agar with phosphate buffer to pH 6.4.

### Cultivation of yeast for RNA isolation.

For RNA isolation, the strains were cultured overnight in SD, harvested by centrifugation, and washed four times in iron-free water. All steps were carried out with acid-washed iron-free equipment. Cells were adjusted to 1 × 10^7^ cells/ml in iron-free citrate-buffered SD (pH 5.8) supplemented with 5 µM FeCl_3_ and grown for 4 h at 37°C and 180 rpm. The 0-h sample was immediately frozen in liquid nitrogen and stored at −80°C. The remaining cultures were harvested by centrifugation and washed four times with iron-free SD. The start of the time series was defined by the first contact with iron-free SD. Further samples were taken at 0.5 h, 1 h, 2 h, and 4 h. To obtain iron-replete samples, 100 µM FeCl_3_ was added following the 4-h time point and a sample was taken after an additional 0.5 h. RNA was isolated with an RNeasy minikit (Qiagen), and quality was verified using an Agilent 2100 Bioanalyzer Nanochip system according to the manufacturer’s protocol. The concentration was determined using a Nanodrop 2000 instrument.

### Microarray.

For microarray analysis, 1 µg high-quality RNA was used to generate cRNA fluorescently labeled with Cy5 CTP (GE Healthcare, United Kingdom) using a one-color Quick Amp labeling kit (Agilent). For a common reference experimental design, cRNA from mid-log-phase YPD-grown *C. glabrata* was labeled with Cy3 CTP (GE Healthcare). After cleanup with an RNeasy minikit (Qiagen), sufficiency of dye incorporation (specific activity, >7 pmol/µg) was checked by NanoDrop. *C. glabrata* arrays were purchased from Agilent Technologies (8-by-15K format) (GEO accession number for the *C. glabrata* Agilent Array, GPL10713 [73]), hybridized using a Gene Expression Hybridization kit (Agilent) as described previously (73), and scanned using a GenePix 4200AL scanner (software, GenePix Pro 6.1; automatically calibrated photomultiplier tube [PMT] wavelengths, 635 and 594 nm; pixel size, 5 µm). Data were extracted with AgilentFE and imported into GeneSpring 12 for data analysis. The experiment was conducted in biological triplicate to cover biological variation. The microarray data are available at the NCBI GEO microarray repository (see below).

### qRT-PCR.

For qRT-PCR, 600 ng high-quality RNA was treated with DNase (Epicentre) and reverse transcribed into cDNA using oligo(dT) primers and Superscript III (Invitrogen). One microliter of cDNA (1:20 dilution) was used for gene expression analysis in a C1000 thermocycler (Bio-Rad; CFX96 Realtime system) using an EvaGreen system (Bio & Sell). The expression rates were determined in biological triplicate and normalized to expression of the housekeeping genes *EFB1* and *EFT2* using Bio-Rad CFX Manager 3.1. All primers are listed in Dataset S5 in the supplemental material.

### Determination of cellular iron content.

For the determination of cellular iron content, a modified ferrozine-based colorimetric method (74) was used. *C. glabrata* cells grown overnight in YPD were inoculated into fresh YPD medium and grown to logarithmic phase (OD_600_, ~1). From that, 100 ml was harvested, washed with iron-free water, resuspended in 600 µl 0.5 M NaOH, and disrupted after addition of 300 mg iron-free acid-washed glass beads using a Precellys cell homogenizer (6,500 × *g*; 3 homogenizations at 45 s per homogenization with a 15-s pause between homogenizations). Beads and cell debris were removed by centrifugation, and 200 µl of the cell supernatant or 0.5 M NaOH (negative control) was used for detection. A 1:2 serial dilution (300 µM to 4.68 µM) of FeCl_3_–0.5 M NaOH was used as a standard. To free heme-bound iron, 100 µl freshly prepared iron-releasing agent (equal volumes of 1.4 M HCl and 4.5% [wt/vol] KMnO_4_) was added to the samples, followed by incubation for 2 h at 60°C and 300 rpm and cooling for 5 min with open lids. Finally, 30 µl of detection mix (6.5 mM ferrozine, 6.5 mM neocuproine to suppress copper signals, 2.5 M ammonium acetate, 1 M ascorbic acid; prepared fresh in iron-free water) was added and samples were incubated for 30 min at room temperature. The iron content was determined by measurement of the OD_568_ normalized against the cell number (initial OD_600_). The experiment was performed in biological triplicate.

### Whole-blood killing assay.

Venous blood of healthy volunteers was collected in hirudin Monovettes (Sarstedt). Overnight *C. glabrata* YPD cultures were harvested and washed twice with sterile water. Yeasts (1 × 10^6^ in 10 µl) were added to 990 µl freshly drawn human blood and incubated using gentle rolling at 37°C. The initial inoculum (0 h) was determined, and after 30, 60, 120, and 240 min, 10 µl of infected blood sample was diluted and plated onto YPD agar (or SD supplemented with 500 µM in FeCl_3_ for the *aft1*Δ mutant) for CFU determination in technical triplicate. This assay was performed independently with three healthy blood donors.

### *In silico* analysis and statistics.

Information about gene orthologs and intergenic regions was obtained from the Candida Genome Database (CGD—http://www.candidagenome.org), the *Candida* Gene Order Browser (CGOB—http://cgob.ucd.ie/), and the Saccharomyces Genome Database (SGD—http://www.yeastgenome.org/). GO terms were obtained from the CGD and processed using the online tool Revigo (http://revigo.irb.hr/). GO terms with a negative log_10_ *P* value higher than 3 (*P* < 0.001) are shown. The 3′ UTR of ORFs was predicted as described by Linde et al. (75) and downloaded from CGD. MEME (37) (http://meme-suite.org) was used to identify enriched motifs in a list of genes or to find genes carrying a target motif via MEME/FIMO (http://meme-suite.org/tools/fimo). For analysis of statistics, GraphPad Prism 5 (GraphPad Inc.) was used. All data are reported as means ± standard errors of the means (SEM) or standard deviation where appropriate, and the two-tailed, unpaired Student’s *t* test was performed, if not stated otherwise. Similarity (nonsignificant differences) of variance data was tested in Prism 5 for all tests. Statistically significant results were marked as follows: *, *P* < 0.05; **, *P* < 0.01; ***, *P* < 0.001.

### Ethics statement.

Human peripheral blood was collected from healthy volunteers after written informed consent was obtained. The study was conducted in accordance with the Declaration of Helsinki, and all protocols were approved by the Ethics Committee of the University Hospital Jena (permission number 2207-01/08).

### Accession number(s).

The microarray data determined in this work are available at the NCBI GEO microarray repository (accession number GSE84816).

## SUPPLEMENTAL MATERIAL

Dataset S1  Iron screen results for all tested *C. glabrata* mutants. Download Dataset S1, XLSX file, 0.2 MB

Dataset S2  *C. glabrata* genes with ScAft1-binding motif PyPuCACCCPu in 5′ intergenic region (IR) identified by FIMO. Download Dataset S2, XLSX file, 0.1 MB

Dataset S3  Expression analysis of iron-associated target genes by qRT-PCR under iron-replete conditions. Data represent expression of target genes for the WT, *cth2*Δ, and *sef1*Δ strains at 0 h and 4 h (under iron deprivation conditions) and at +0.5 h (under iron supplementation conditions). After 4 h preincubation under low-iron conditions (50 µM FeCl_3_), the 0-h sample was obtained. Residual cells were then incubated without iron for 4 h before 100 µM FeCl_3_ was supplemented (+0.5 h). Target gene expression was normalized to that of housekeeping genes *EFB1* and *EFT2*. Expression is shown relative to that of the WT at 0 h in biological triplicate experiments (standard deviation ± SEM; n.d., no expression detected). Statistical significance data were determined by an unpaired Student’s *t* test (*, *P* value < 0.05; **, *P* value < 0.01; ***, *P* value < 0.001). (a) Expression of the regulators of iron metabolism *AFT1* and *SEF1* (encoding transcription factors) and *CTH2* (encoding mRNA-degrading protein)*.* (b) Expression of iron acquisition genes *FTR1* (encoding an HA-iron transporter), *SIT1* (encoding a siderophore transporter), *HMX1* (degrading heme oxidase), and *FTH1* (encoding a vacuolar HA-iron exporter). (c) Expression of iron consumption genes *CCP1* (encoding mitochondrial cytochrome c peroxidase), *CYT1* (encoding cytochrome c), *HEM15* (encoding heme synthesis), and *CCC1* (encoding a vacuolar HA-iron importer). (d) Expression of the following genes affected by *SEF1* deletion: *ACO1* (encoding mitochondrial aconitase), *ISA1* (encoding an iron sulfur cluster assembly), and *IDH2* (encoding isocitrate dehydrogenase). Download Dataset S3, EPS file, 2.1 MB

Dataset S4  *C. glabrata* ORFs with Cth2 nonamer recognition motif and degenerated variants in 3′ intergenic region (IR) with FIMO. Download Dataset S4, XLSX file, 0.2 MB

Dataset S5  Primers used in this study. Download Dataset S5, DOCX file, 0.02 MB
