# Supplementary material for: A Novel Hybrid Iron Regulation Network Combines Features from Pathogenic and Nonpathogenic Yeasts
Source: mBio. 2016 Oct 18;7(5):e01782-16. doi: 10.1128/mBio.01782-16 (PMC5082906; doi:10.1128/mBio.01782-16)
Supplement: Dataset S5 — Primers used in this study. Download [file mbo005163042sd5.docx]

**S5 Dataset. Primers used in this study**

| Application context | Name | Sequence (5‘ 🡪 3‘) |
| --- | --- | --- |
| General Primers |  |  |
|  | U1 fwd | CCGCTGCTAGGCGCGCCGTG |
|  | D1 rev | GCAGGGATGCGGCCGCTGAC |
|  | puC19 MCS fwd | GGCGATTAAGTTGGGTAACG |
|  | puC19 MCS rev | TGGAATTGTGAGCGGATAAC |
|  | NAT up rev | GAACCATCCAAAGCTTCAATAG |
|  | NAT dn fwd | TTGTGGTTGGAAGTTACCAATG |
|  | NAT fwd South | CTGTTCCAGGTGATGCTGAAG |
|  | NAT rev South | CGAATTCAGTAGCCAAACCCAT |
| Gene KO primers |  |  |
|  | Aft1-fwd-pUC19 | GCAGGTCGACTCTAGGCAGGAACTTATACGTTGAAATTAC |
|  | Aft1- rev-PUC19 | CCGGGGATCCTCTAGGCAGAGAAGGAAAGATACAAGTATG |
|  | Aft1-U1-flank-BC | CGCGCCTAGCAGCGGGCAGGTAATATCTTGATTTTCTTTC |
|  | Aft1- D1-flank-BC | CGGCCGCATCCCTGCCACATGGTAAACTTTAACCCAAGAG |
|  | Aft1-fwd-5'UTR | TCGTTCCCTGCATGCGAACAAG |
|  | Aft1-rev-3'UTR | TTCCGAGGTCTGACCGTGAATG |
|  | Aft1-fwd | GCAGGAACTTATACGTTGAAATTAC |
|  | Aft1-rev | GCAGAGAAGGAAAGATACAAGTATG |
|  | Ftr1-fwd-pUC19 | GCAGGTCGACTCTAGATTTAGTGAAGTAGTGGTGCTATCC |
|  | Ftr1- rev-PUC19 | CCGGGGATCCTCTAGCAATACCAGGCATATCCCTGAGAAC |
|  | Ftr1-U1-flank-BC | CGCGCCTAGCAGCGGTAGCCAGGTTGACCTTTACCATTTC |
|  | Ftr1- D1-flank-BC | CGGCCGCATCCCTGCTCCAATATCACATGGGTTCCCAAAG |
|  | Ftr1-fwd-5'UTR | CCATGTACCACCAATTGTAACG |
|  | Ftr1-rev-3'UTR | TGTTTCGTTCTTCCGGCTAACC |
|  | Ftr1-fwd | ATTTAGTGAAGTAGTGGTGCTATCC |
|  | Ftr1-rev | CAATACCAGGCATATCCCTGAGAAC |
|  | Fet4-fwd-pUC19 | GCAGGTCGACTCTAGTAGTGTTCGCATCAGCAATAACTTC |
|  | Fet4- rev-PUC19 | CCGGGGATCCTCTAGCCGCTGTGATACTCTTTAACGTTTG |
|  | Fet4-U1-flank-BC | CGCGCCTAGCAGCGGGTAGATGAATTAGAATTGCGCTCTC |
|  | Fet4- D1-flank-BC | CGGCCGCATCCCTGCAGAGGCTGCTTATTAAGCTTCTCAC |
|  | Fet4-fwd-5'UTR | CAGTGCTGCATTCAAGGTCAAG |
|  | Fet4-rev-3'UTR | ATCGCTGCCTCAATTTGTTGTG |
|  | Fet4-fwd | TAGTGTTCGCATCAGCAATAACTTC |
|  | Fet4-rev | CCGCTGTGATACTCTTTAACGTTTG |
|  | Fet3-fwd-pUC19 | GCAGGTCGACTCTAGGTTGTTGTAACTTTGACTGGCATTG |
|  | Fet3- rev-PUC19 | CCGGGGATCCTCTAGTTCCAAGAGTTGGCTAATATCAATG |
|  | Fet3-U1-flank-BC | CGCGCCTAGCAGCGGGTGGATCAGGATTTGTGCTCATAAG |
|  | Fet3- D1-flank-BC | CGGCCGCATCCCTGCAACTGGAGGTTTAATGGTTCTAGAG |
|  | Fet3-fwd-5'UTR | GGGAAAGCCGACAAAGAACTAC |
|  | Fet3-rev-3'UTR | TGGCACGTTTACAATCACCTTC |
|  | Fet3-fwd | GTTGTTGTAACTTTGACTGGCATTG |
|  | Fet3-rev | TTCCAAGAGTTGGCTAATATCAATG |
|  | Hap4-fwd-pUC19 | GCAGGTCGACTCTAGAAATCATGGTCCTTACCTTGTGAAC |
|  | Hap4-rev-pUC19 | CCGGGGATCCTCTAGCTACAGTGGTGGAGTAGGTATATTC |
|  | Hap4-U1-flank-BC | CGCGCCTAGCAGCGGGAGTTAGCGGTATAAGGTACCATTG |
|  | Hap4-D1-flank-BC | CGGCCGCATCCCTGCGAAGCCTAGCCTATTTATGCTTGTC |
|  | Hap4-fwd-5'UTR | CTGCGCATGTTCCTCTCAATAC |
|  | Hap4-rev-3'UTR | TGACCTGCTGGACATCCATATC |
|  | Hap4-fwd | AAATCATGGTCCTTACCTTGTGAAC |
|  | Hap4-rev | CTACAGTGGTGGAGTAGGTATATTC |
|  | Hap5-fwd-pUC19 | GCAGGTCGACTCTAGCTATTTCTCCGCCTCGTTATATCTC |
|  | Hap5- rev-pUC19 | CCGGGGATCCTCTAGCCCAACATTCGCACTACTATTACAC |
|  | Hap5-U1-flank-BC | CGCGCCTAGCAGCGGCAGTACTCTGCCTTTGTAACTATTC |
|  | Hap5- D1-flank-BC | CGGCCGCATCCCTGCGATGTCCTGTACGGGTTTACAAGTG |
|  | Hap5-fwd-5'UTR | TGTCGCCATGCAAAGTAATTAG |
|  | Hap5-rev-3'UTR | TCGCATTTCCAACAGTTGTATC |
|  | Hap5-fwd | CTATTTCTCCGCCTCGTTATATCTC |
|  | Hap5-rev | CCCAACATTCGCACTACTATTACAC |
|  | Cth2-fwd-pUC19 | GCAGGTCGACTCTAGTCTCTTCGACTTCTATGCTGATCAC |
|  | Cth2- rev-pUC19 | CCGGGGATCCTCTAGTCACCCAAGTGGTCTATATTACAAG |
|  | Cth2-U1-flank-BC | CGCGCCTAGCAGCGGTTGATACTTATGCTTGTGCTAAGTC |
|  | Cth2- D1-flank-BC | CGGCCGCATCCCTGCGCTGATTATCAGTTCTAATCTGAAG |
|  | Cth2-fwd-5'UTR | TCTCCTTGATGGAGGATATCAG |
|  | Cth2-rev-3'UTR | TATACTGGTCCGAAACCAATCC |
|  | Cth2-fwd | TCTCTTCGACTTCTATGCTGATCAC |
|  | Cth2-rev | TCACCCAAGTGGTCTATATTACAAG |
|  | sef1 na fwd | CGCCATGCACCATACAGAATAC |
|  | sef1 na rev | AATGACGTAGGGAGCAATTCTG |
|  | Sef1 na CoP1 fwd | AGTCAAGTCCTACCTACGTTAC |
|  | Sef1 na CoP4 rev | CAGATGAATCCACAGACAAATG |
| qRT-PCT primers |  |  |
|  | EFB1-RT-P1 | GACACTGTCAAGGAATTGAACAC |
|  | EFB1-RT-P2 | GAAGCAGCTGGGAAGGAGT |
|  | qRT EFT2 HK fwd | TACTCTAAACTCCGACCCATTG |
|  | qRT EFT2 HK rev | TCCTTCATACCGTGTCTCTTAC |
|  | qRT FTR1 fwd | AAGAAGAAGGACGAATTGACTG |
|  | qRT FTR1 rev | TTCTTCCTTGGATCCCATCTTG |
|  | qRT SIT1 fwd | TGGGTCTACTATACCACTACAG |
|  | qRT SIT1 rev | ACAGATACGTTAACTGGGTAAG |
|  | qRT AFT1 fwd | GAGCAAAGGTCAGATTCTTTAC |
|  | qRT AFT1 rev | AATTCATCTCCAACGTCATGTC |
|  | qRT FTH1 fwd | AAGAGCAATACGGTTACATTCC |
|  | qRT FTH1 rev | GGGTAGTGCTATCCTTTGAAAC |
|  | qRT SEF1 fwd | AGATAGACCAAACAGTGCTATG |
|  | qRT SEF1 rev | TTGCTGAGTAGAAGAGTTTACG |
|  | qRT CTH2 fwd | ACAAGCAAGTTTCACGTAAGTC |
|  | qRT CTH2 rev | GAAGTTAGTGGAAGCGCATTAG |
|  | qRT HMX1 fwd | CGTCATGTACCTCGCTTTATTC |
|  | qRT HMX1 rev | GAACAAGCCAGTGTTCCTGTAG |
|  | qRT CCC1 fwd | TGGACTTCATCATTAGGTATGG |
|  | qRT CCC1 rev | TGACCACGATTGAGTATATCAG |
|  | qRT IDH1 fwd | CACGTCGGTCTAGATATCAAGG |
|  | qRT IDH1 rev | TTCTCTTAGCGGATTCTGTTAG |
|  | qRT FET4 fwd | CGGTGTTGTGGTAGCTATTATC |
|  | qRT FET4 rev | TAAACCGGTGTAAGTACCAATG |
|  | qRT FET3 fwd | TATCCCAACAGGTTTCACAAAG |
|  | qRT FET3 rev | TGATCTCTGGTTCTGGGATTTC |
|  | qRT HEM15 fwd | TGAAATTGGTAAGGAGGTCATC |
|  | qRT HEM15 rev | AATACAGGACTTCCGTTCAATG |
|  | qRT CYT1 fwd | CACCAACTACAACCCATACTTC |
|  | qRT CYT1 rev | CTTCAAACCTGCCTTCTTTCTC |
|  | qRT CYC1 fwd | CACCAACTACAACCCATACTTC |
|  | qRT CYC1 rev | CTTCAAACCTGCCTTCTTTCTC |
|  | qRT CCP1 fwd | TGCTTTGGGTAAGACACATTTG |
|  | qRT CCP1 rev | GTCTTGGATCAAAGCGTAATCG |
|  | qRT ERG11 fwd | CGATACCAAGGAATTGACTTAC |
|  | qRT ERG11 rev | GTGTTTGGAATAGCGACATCTC |
|  | qRT MMT2 fwd | AACTCTTGCAAAGCTTGTAACC |
|  | qRT MMT2 rev | CGTTGGAGAGGTACTGTTAGAG |
|  | qRT COX6 fwd | GAGAGCTGCTAGAAGAGTCAATG |
|  | qRT COX6 rev | GAGGAACACCCAATTCTTCTC |
|  | qRT ISA1 fwd | TTCCATACCAGACATACGAAAC |
|  | qRT ISA1 rev | CCTTAACTGGTACTGCCATTG |
|  | qRT IDH2 fwd | AAAGAACTAGCAGCAGAGTACC |
|  | qRT IDH2 rev | TGACAAGATGTCACCGTACAAG |
|  | qRT ACO1 fwd | TCTACAAGTCCTAAAGCCTTTC |
|  | qRT ACO1 rev | GCACCAATCATGTAGTTGTTAG |

**Underlined: primer overhangs**

**Grey: homolog to *Xba*1 linearized vector pUC19**

**Green: homolog to *NAT1* cassette flanking regions (U1 or D1)**
